# High Pretransplant BAFF Levels and B-cell Subset Polarized towards a Memory Phenotype as Predictive Biomarkers for Antibody-Mediated Rejection

**DOI:** 10.3390/ijms21030779

**Published:** 2020-01-25

**Authors:** Juan Irure-Ventura, David San Segundo, Emilio Rodrigo, David Merino, Lara Belmar-Vega, Juan Carlos Ruiz San Millán, Rosalía Valero, Adalberto Benito, Marcos López-Hoyos

**Affiliations:** 1 Immunology Department. University Hospital Marqués de Valdecilla-IDIVAL, 39008 Santander, Spain; juan.irure@scsalud.es (J.I.-V.); david.sansegundo@scssalud.es (D.S.S.); 2 Nephrology Department. University Hospital Marqués de Valdecilla-IDIVAL, 39008 Santander, Spain, lara.belmar@scsalud.es (L.B.-V.); juancarlos.ruiz@scsalud.es (J.C.R.S.M.); rosalia.valero@scsalud.es (R.V.); 3 Health Research Institute-IDIVAL, 39011 Santander, Spain; david.merino@hotmail.com (D.M.); adalberto.benito@scsalud.es (A.B.)

**Keywords:** antibody-mediated rejection, kidney transplantation, BAFF, B cell subpopulations, non-invasive biomarker

## Abstract

Antibody-mediated rejection (AbMR) is one of the leading causes of graft loss in kidney transplantation and B cells play an important role in the development of it. A B-cell activating factor (BAFF) is a cytokine involved in B cell ontogeny. Here, we analyzed whether B cell maturation and the effect of B cell soluble factors, such as BAFF could be involved in AbMR. Serum BAFF levels and B and T cell subpopulations were analyzed 109 kidney transplant patients before transplantation and at 6 and 12 months after kidney transplantation. Pretransplant serum BAFF levels as well as memory B cell subpopulations were significantly higher in those patients who suffered clinical AbMR during the first 12 months after kidney transplantation. Similar results were observed in the prospective analysis of patients with subclinical antibody-mediated rejection detected in the surveillance biopsy performed at 12 months after kidney transplantation. A multivariate analysis confirmed the independent role of BAFF in the development of AbMR, irrespective of other classical variables. Pretransplant serum BAFF levels could be an important non-invasive biomarker for the prediction of the development of AbMR and posttransplant increased serum BAFF levels contribute to AbMR.

## 1. Introduction

The importance of B cells in kidney transplantation, especially in the case of antibody-mediated rejection (AbMR) and transplantation tolerance, has been highlighted in recent studies [1,2,3]. It is well known the crucial role of B cells in humoral immunity, but they also contribute in other important processes, such as co-stimulation, antigen presentation, and cytokine secretion, all of them mechanisms that modulate the function of T cells [4]. AbMR is considered the main cause of kidney transplant failure [5,6,7]. AbMR is characterized by histological findings, like microvasculature inflammation, anti-HLA antibodies, mainly donor-specific antibodies (DSA), and C4d deposition in renal tissue [8]. However, the mechanisms underlying these processes are not well understood, and therefore, the analysis of different B cell subpopulations and cytokines associated with B cell activation and survival could allow us to improve the knowledge of the pathogenesis, and to predict the allograft outcome.

The B cell homeostasis is modulated by different soluble factors, mainly the B-cell activating factor (BAFF, also known as TNFSF13B or the B lymphocyte stimulator, BLyS) and proliferation-inducing ligand (APRIL). BAFF belonging to the TNF family is a cytokine expressed and secreted by several cells of the immune system, predominantly myeloid cells (monocytes, macrophages, and dendritic cells), neutrophils, and by a subset of T lymphocytes, and is involved in survival, proliferation, differentiation, and maturation of different B cell subpopulations [9,10]. It is able to bind to three receptors, BAFF receptor (BAFFR, also known as TNFRSF13C or BLyS receptor 3), T cell activator and calcium modulating ligand interactor (TACI), and B cell maturation antigen (BCMA). The three receptors are expressed in B cells at different stages of development, being BAFFR the first one to be expressed and the only one required for survival of transitional and naïve B cells. TACI is expressed in B cells upon activation, whereas BCMA is found in germinal center B cells and in terminally differentiated B cells [11].

It has been analyzed that defects in the expression of either BAFF or BAFFR impairs B cell development beyond the immature, transitional type 1 stage and thus, prevents the formation of follicular and marginal zone B cells [12,13]. In the same way, experiments in mice have shown that defects in B cells receptor and BAFFR signaling entail an absence of mature peripheral B cell populations [14]. Thaunat et al. found that BAFF provides survival signals to B cells and allows them to escape rituximab-induced apoptosis in tertiary lymphoid organs [15].

Several studies have demonstrated the important role of BAFF in B cell biology, enhancing humoral immune responses to both T cell dependent and independent antigens, inducing class switch recombination and also, acting as a co-stimulator of T cell activation [16,17]. Furthermore, it is well established the implication of BAFF in the pathogenesis of different autoimmune diseases, such as systemic lupus erythematosus [18], Sjögren’s syndrome [19], rheumatoid arthritis [20], systemic sclerosis [21], or multiple sclerosis [22], among others.

However, in the context of transplantation, there are less studies and the role of BAFF is more controversial. On the one hand, some authors indicate that BAFF reflects the immunological risk profile of patients after kidney transplantation (KT), considering that pretransplant BAFF levels are associated with pretransplant sensitization and are useful in predicting allograft rejection [23,24,25]; BAFF expression correlates with pretransplant panel reactive antibody (PRA), indicating that BAFF may be involved in the development of graft loss [26]; elevated levels of BAFF are associated with antibody-mediated clinical damage in KT [27], and with an increased risk of acute AbMR [28,29,30]. On the other hand, there are authors that affirm that BAFF is not a prognostic marker for allograft dysfunction or survival in KT patients because BAFF serum levels are not related to anti-HLA sensitization [31]; significantly lower levels of BAFF are found in patients experiencing AbMR [32]; or high BAFF is not associated with the graft outcome in KT with rituximab induction [33]. A meta-analysis published recently evaluated the predictive value of serum BAFF for AbMR, indicating that the incidence of AbMR was significantly higher in patients with high levels of BAFF [34].

Another important aspect is the relationship between B and T cells, specifically CD4^+^ T cells, which are crucial for the generation of functional B cells. Unlike naïve T cells, memory T cells are able to activate in the presence of lower costimulatory signals. Following activation, memory T cells proliferate less than primary responding cells but can produce a greater effector response [35]. However, the different expression of T cell subpopulations and their relationship with B cells is not well understood in KT patients.

Taking into account these circumstances, in this study we analyzed the role of pretransplant BAFF levels, as well as at different time points after KT in the development of AbMR, evaluating together the changes that occur in the distribution of different B and T cell subpopulations in these KT patients.

## 2. Results

### 2.1. Pretransplant Serum BAFF Levels in Kidney Transplant Patients

**Figure 1:**
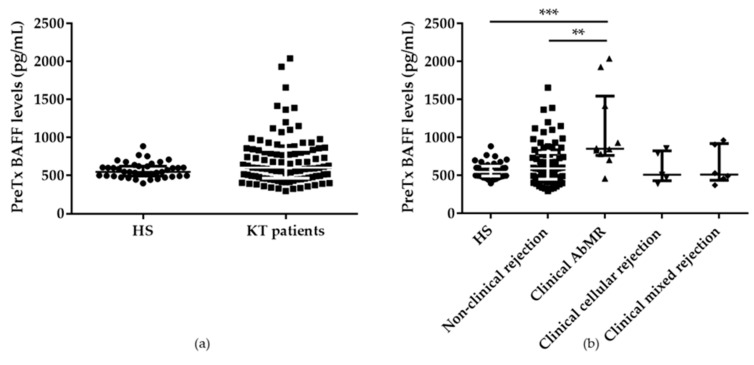
(**a**) No significant differences were found in the comparison of pretransplant serum BAFF levels between kidney transplant (KT) patients (*n* = 109) and healthy subjects (HS; *n* = 40). (**b**) Statistically significant differences in pretransplant serum BAFF levels between kidney transplant patients considering the different types of clinical rejection (non-clinical rejection (*n* =87), antibody-mediated rejection (*n* = 11), cellular rejection (*n* = 5), and mixed rejection (*n* = 6)) and healthy subjects. ** Indicates *p* < 0.01, and *** *p* < 0.001

Pretransplant serum BAFF levels were higher in KT patients (604.63; IQR 465.18-845.42 pg/mL) than in healthy subjects (HS; 549.88; IQR 495.43-622.97 pg/mL) without reaching statistical significance. However, when KT patients were stratified according to the presence of clinical rejection during the first 12 months after KT, patients who suffered clinical AbMR presented significantly higher pretransplant serum BAFF levels (853.29; IQR 765.37-1545.99 pg/mL) than KT without clinical rejection (594.60; IQR 453.21-803.93 pg/mL) or HS, *p* = 0.003 and *p* < 0.001, respectively (Figure 1a,b).

Furthermore, significantly higher pretransplant serum BAFF levels were observed in those patients that presented pretransplant anti-HLA antibodies (836.22; IQR 594.11–1140.75 pg/mL vs. 568.25; IQR 448.23–778.01 pg/mL) and DSA (1153.09; IQR 914.34–1405.35 pg/mL vs. 601.64; IQR 462.72–833.26 pg/mL), *p* = 0.001 and *p* = 0.049, respectively. A positive association was observed between BAFF levels and pretransplant calculated panel-reactive antibodies (cPRA; r = 0.355, *p* < 0.001). When we analyzed the presence of anti-HLA antibodies at 6 months posttransplantation, significantly higher pretransplant BAFF levels were found in this group of patients, *p* = 0.001.

**Figure 2:**
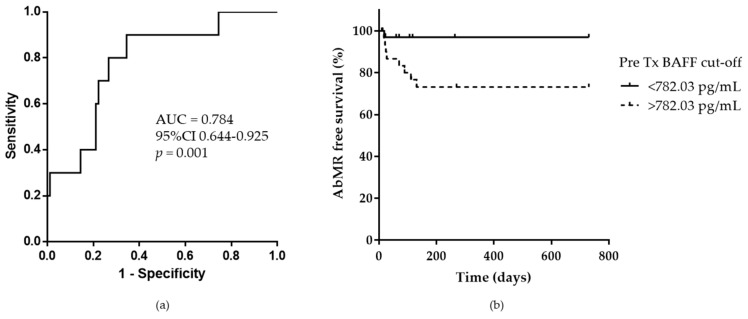
(**a**) Receiver operational curve analysis of BAFF levels before transplantation and the development of antibody-mediated rejection (AbMR) during the first 12 months after kidney transplantation. The area under curve (AUC) is 78.4%. Based on Youden’s index, a cut-off of 782.03 pg/mL pretransplant BAFF levels discriminate between clinical AbMR and non-rejection in the first 12 months after kidney transplantation with a sensitivity and specificity of 80% and 73.3%, respectively. (**b**) AbMR free survival between patients with BAFF levels higher and lower than 782.03 pg/mL.

Area under the receiver operational curve (ROC) of pretransplant soluble BAFF for predicting clinical AbMR during the first 12 months after kidney transplantation was 0.784 (95% CI 0.644–0.925). Based on this analysis and applying Youden’s index, the optimal cut-off point for distinguishing between low and high BAFF levels was 782.03 pg/mL, with high sensitivity and specificity at 80% and 73.3%, respectively. Considering this cut-off point, KT patients with pretransplant serum BAFF levels higher than 782.03 pg/mL, showed statistically significant less AbMR free survival (log-rank *p* < 0.001; Figure 2a,b).

Cox regression analysis confirmed a significant, independent role of pretransplant serum BAFF levels in the development of AbMR during the first 12 months after KT, HR 1.002; 95% CI 1.001–1.003, *p* = 0.008. Covariates included in the analysis were pretransplant anti-HLA antibodies, pretransplant DSA, a number of previous transplants, total HLA (HLA-A, -B, -C, -DRB1, and -DQB1) mismatches, induction therapies, cold ischemia time (CIT), and delayed graft function (DGF). A high level of serum BAFF was a risk factor for the development of the event, irrespective of classical variables. An increase of 100 pg/mL of BAFF levels increased 1.35 times the risk of AbMR. When Cox regression analysis was performed after stratifying KT patients according to the cut-off of 782.03 pg/mL, pretransplant serum BAFF levels confirmed their independent role in the development of AbMR in the first 12 months after KT, HR 6.945; 95% CI 1.415–34.082, *p* = 0.017, indicating that patients with pretransplant serum BAFF levels higher than 782.03 pg/mL presented around seven times more risk of developing AbMR.

Similar results were obtained in the multivariate regression analysis in order to determine the independent role of pretransplant serum BAFF levels in the development of AbMR during the first 12 months after KT, HR 1.003; 95% CI 1.001–1.005, *p* = 0.004. The same covariates as in Cox regression analysis were included.

### 2.2. Pretransplant B Cell Subpopulations Distribution in AbMR Patients

First of all, we performed an extensive immunophenotyping of pretransplant B and T cell repertoires of KT recipients considering the presence of clinical AbMR compared with 40 HS. As in other publications, KT patients showed a significant B-cell depletion that resulted in a reduction of absolute numbers and percentages of pretransplant B cells in the KT group compared with HS. However, absolute B cell numbers of KT patients that developed a clinical AbMR did not differ from stable KT patients.

**Figure 3:**
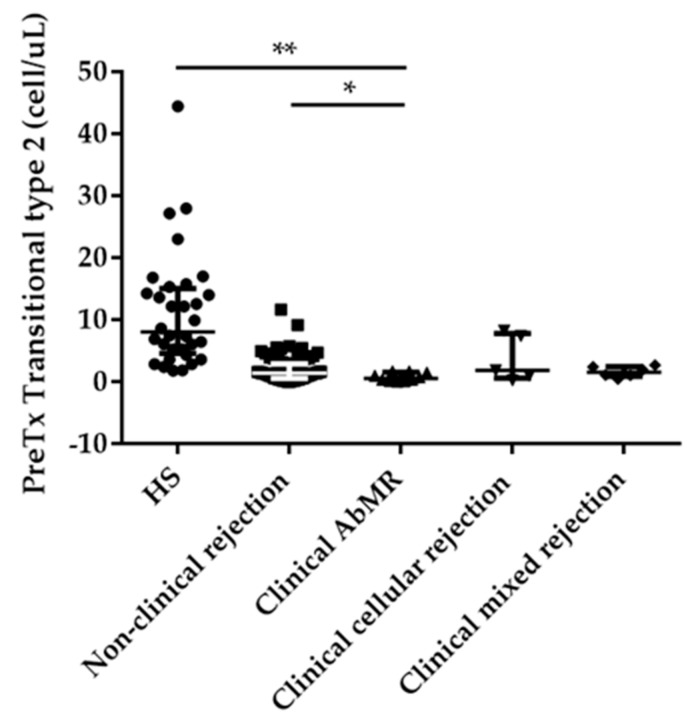
Significant reduction in the absolute number of pretransplant transitional type 2 cells in antibody-mediated rejection (AbMR; *n* = 11) when compared with the non-rejection group (*n* = 87) and healthy subjects (HS; *n* = 40). * indicates *p* < 0.05, and ** *p* < 0.01

Analyzing the naïve B cell stage, we found that KT patients who suffered clinical AbMR showed a significant reduction in absolute numbers and percentages of transitional B cells, specifically type 2 (T2), which is characterized by the expression of CD5^low^ when compared with stable KT patients (0.52; IQR 0.11–1.51 cells/µL vs. 1.35; IQR 0.54–2.88 cell/µL and 0.15; IQR 0.09–0.92% vs. 1.34; IQR 0.40–2.57%, *p* = 0.039 and *p* = 0.003, respectively), and with HS (Figure 3). 

**Figure 4:**
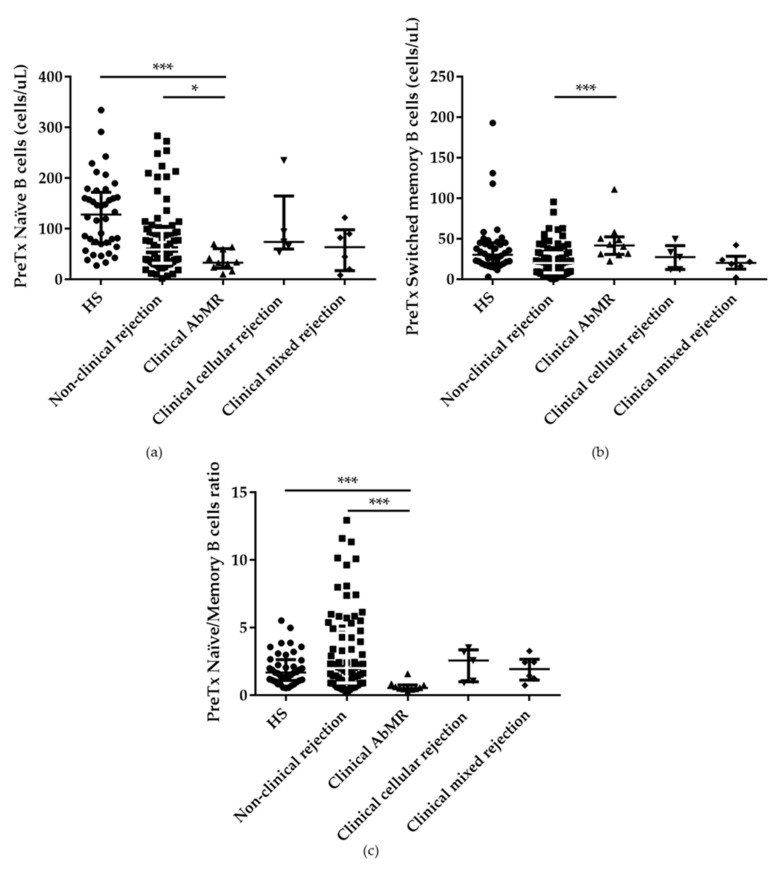
Abnormal ratio between pretransplant naïve and memory B cells. (**a**) Significant reduction of absolute numbers of naïve B cells in antibody-mediated rejection (AbMR) patients (*n* = 11). (**b**) Higher numbers of switched memory B cells in patients that developed AbMR. (**c**) Reduction of pretransplant naïve/memory B cells ratio between AbMR patients and non-clinical rejection group (*n* = 87) and healthy subjects (HS; *n* = 40). * indicates *p* < 0.05, and *** *p* < 0.001

Besides, we observed that AbMR patients showed an abnormal distribution of other pretransplant B-cell subpopulations, such as naïve (CD19^+^ IgD^+^ CD27^-^) and switched memory (CD19^+^ IgD^-^ CD27^+^) B cells. These patients presented significantly lower levels of naïve B cells (33.65; IQR 22.60–61.31 cells/µL) when compared with KT patients without clinical rejection (59.61; IQR 32.39–97.07 cells/µL), and with HS (128.54; IQR 71.13–172.53 cells/µL), *p* = 0.04 and *p* < 0.001, respectively. On the other hand, patients with AbMR had significantly higher levels of switched memory B cells (41.93; IQR 31.03–52.53 cells/µL), than the rejection-free group (20.39; IQR 8.28–32.87 cells/µL) or HS (30.42; IQR 20.18–45.38 cells/µL), *p* = 0.001 and *p* = 0.11, respectively (Figure 4a,b). Considering the ratio between naïve and memory (switched + unswitched) B cells, this ratio is reduced in patients who suffered AbMR in comparison with stable patients and healthy volunteers, *p* < 0.001 in both cases (Figure 4c).

**Figure 5:**
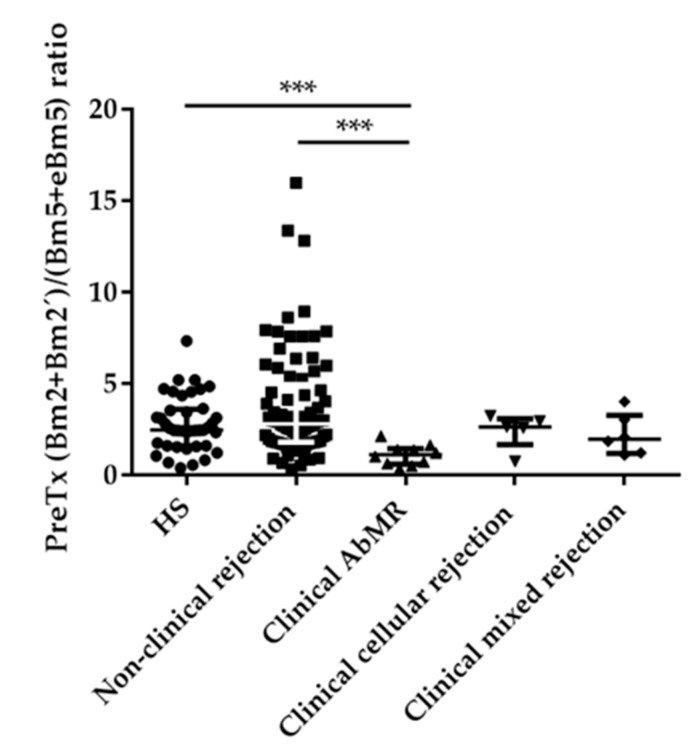
Predominance of pretransplant memory B cells subpopulations (Bm5 + eBm5) in antibody-mediated rejection (AbMR; *n* = 11) in comparison with non-rejection group (*n* = 87) and healthy subjects (HS; *n* = 40). *** indicates *p* < 0.001

Similar results were obtained when the B cell compartment was analyzed using the Bm1–Bm5 classification in order to identify different developmental stages from naïve to memory B cells [36]. Bm2 and Bm2’ subpopulations corresponded to activated B cells, whereas Bm5 and eBm5 subpopulations grouped memory B cells. As we have seen before, patients with AbMR presented lower levels of activated B cells (Bm2 + Bm2’; 36.07; IQR 16.66–69.21 cells/µL) than non-rejection KT group (61.32; IQR 36.96–92.81 cell/µL) or HS (105.21; IQR 60.42–161.93 cells/µL), *p* = 0.045 and *p* = 0.001, respectively. On the contrary, AbMR patients showed higher levels of memory B cells (Bm5 + eBm5) compared with non-rejection group (*p* = 0.05) and HS (*p* = ns). The ratio (Bm2 + Bm2’)/(Bm5 + eBm5) was significantly reduced in patients with AbMR (1.12; IQR 0.61–1.46 cells/µL) compared with non-rejection KT patients (2.80; IQR 1.80–4.96 cells/µL) and healthy controls (2.47; IQR 1.60–3.61 cells/µL), *p* < 0.001 in both comparisons (Figure 5). 

To assess the independent role of pretransplant BAFF levels and other B cell subpopulations that showed significant results when they were compared between AbMR and the non-rejection group in the development of clinical AbMR during the first 12 months after kidney transplantation, a multivariate regression analysis was performed including variables classically involved in this process, such as pretransplant anti-HLA antibodies, pretransplant DSA, a number of previous transplants, total HLA (HLA-A, -B, -C, -DRB1, and -DQB1) mismatches, induction therapies, CIT, and DGF. BAFF levels (HR 1.006, 95% CI 1.001–1.011, *p* = 0.014), switched memory B cells (HR 1.050, 95% CI 1.002–1.105, *p* = 0.065) and naïve/memory B cells ratio (HR 0.03, 95% CI 0.001–0.706, *p* = 0.03) were independently associated with AbMR, irrespectively of other classical variables. The higher the BAFF levels and the number of switched memory B cells, the higher the risk of AbMR. Conversely, an increase in the naïve/memory B cells ratio was a protective factor for the development of the clinical event. Collinearity was tested for switched memory B cells and naïve/memory B cells ratio in order to be included in the analysis (*p* = 0.775).

### 2.3. Pretransplant T cell Subpopulations Distribution in AbMR Patients

T cell compartment was also studied as a result of the cooperation between B and T cells, where T cell responses can be influenced by the activity of B cells through co-stimulation signaling and cytokine production. The absolute numbers of CD4^+^ T cells were reduced in patients with AbMR compared with HS. However, the percentages were similar between patients with AbMR, non-rejection KT patients, and HS. When the different T cell subpopulations (naïve, central memory, effector memory, and TEMRA) were compared, patients with AbMR presented a depletion of CD4^+^ naïve T cells (29.99; IQR 15.49%–38.78%) with respect to non-rejection patients (42.47; IQR 23.46%–59.70%) and healthy controls (58.32; IQR 39.66%–66.58%), *p* = 0.038 and *p* < 0.001, respectively. Conversely, higher levels of CD4^+^ TEMRA T cells were observed in these patients (12.90; IQR 4.71%–17.95%) in comparison with stable patients (3.29; IQR 1.40%–7.78%) and HS (0.50; IQR 0.26%–1.11%), *p* = 0.003 and *p* < 0.001, respectively.

Similar results were found when CD8^+^ T cell subpopulations were studied, KT patients who suffered a clinical AbMR showed a reduction of CD8^+^ naïve T cells and a predominance of CD8^+^ TEMRA T cells.

**Figure 6:**
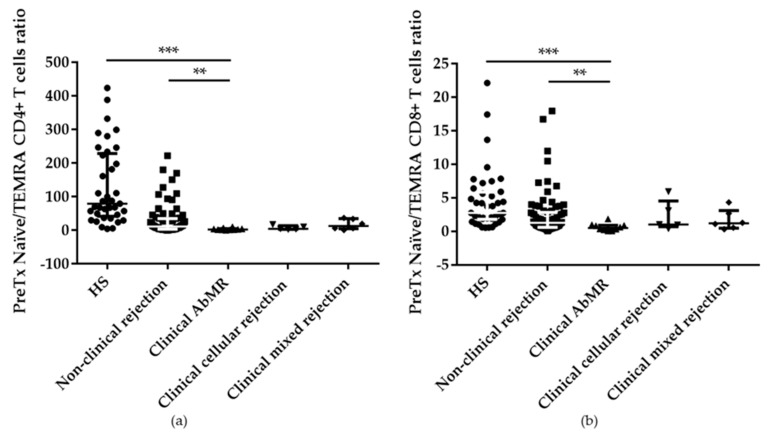
Predominance of a preactivated state in antibody-mediated rejection (AbMR) patients (*n* = 11) in comparison with non-rejection group (*n* = 87) and healthy subjects (HS; *n* = 40), reflected off by a reduction of pretransplant naïve/TEMRA CD4+ (**a**) and CD8+ (**b**) T cells ratio. ** indicates *p* < 0.01, and *** *p* < 0.001

Considering this different distribution of T cells subpopulations in AbMR patients, a polarization towards a preactivated state was observed in this group of patients, since the naïve/TEMRA ratio was significantly diminished in AbMR patients when compared with non-rejection group and HS, *p* = 0.002 and *p* < 0.001, respectively in CD4+ T cells and *p* = 0.006 and *p* < 0.001, respectively in CD8+ T cells (Figure 6a,b).

### 2.4. Serum BAFF Levels at 6 and 12 Months Posttransplantation in Subclinical AbMR Patients

**Figure 7:**
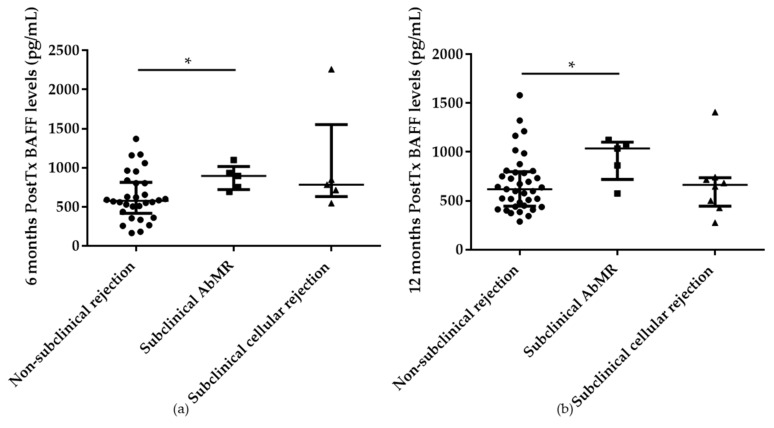
Higher BAFF serum levels at 6 (**a**) and 12 months (**b**) after kidney transplantation in patients with subclinical antibody-mediated rejection (AbMR; *n* = 5) detected retrospectively in the surveillance biopsy performed at 1 year after kidney transplantation, in comparison with non-rejection group (*n* = 38). * indicates *p* < 0.05.

For the prospective study, the 51 selected patients were included (see Material and Methods). Patients who suffered subclinical AbMR presented higher serum BAFF levels at 6 (895.70; IQR 722.44–1016.02 pg/mL) and 12 months (1035.91; IQR 718.87–1100.12 pg/mL) posttransplantation in comparison with non-rejection patients (577.13; IQR 417.73–812.61 pg/mL and 619.17; IQR 446.38–798.15 pg/mL), *p* = 0.048 and *p* = 0.045, respectively (Figure 7a,b).

Area under the ROC curve of soluble BAFF at 6 and 12 months after KT for predicting subclinical AbMR detected retrospectively in the surveillance biopsy performed at 1 year after KT were 0.754 (95% CI 0.604–0.905) and 0.778 (95% CI 0.587–0.968), respectively. Based on this analysis and applying Youden’s index, the best cut-off point for a discriminate between low and high BAFF levels at 6 months after KT was 734.22 pg/mL, with a sensitivity and a specificity of 80% and 65.7%, respectively. At 12 months after KT the optimal cut-off point of BAFF levels was 835.94 pg/mL, with a sensitivity of 80% and a specificity of 82.2%.

A multivariate regression analysis confirmed the independent role of serum BAFF levels at 12 months after KT in the development of subclinical AbMR detected in the surveillance biopsy performed at 1 year after KT, HR 1.004; 95% CI 1.001–1.008, *p* = 0.026. Covariates included in the analysis were anti-HLA antibodies and DSA at 6 and 12 months after KT, number of previous transplants, total HLA (HLA-A, -B, -C, -DRB1, and -DQB1) mismatches, induction therapies, CIT, and sustained DGF. When the multivariate analysis was performed after stratifying KT patients according to the cut-off of 835.94 pg/mL, serum BAFF levels at 12 months after KT confirmed their independent role in the development of subclinical AbMR, HR 18.5; 95% CI 1.817–188.389, *p* = 0.014, indicating that high levels of serum BAFF at 12 months after KT increase the risk of developing AbMR.

In order to establish BAFF serum levels as a useful biomarker for evaluating renal function and predicting the development of AbMR a positive correlation was observed between the albumin/creatinine ratio at surveillance biopsy and serum BAFF levels at 6 months (*r* = 0.326, *p* = 0.04) and at 12 months (*r* = 0.340, *p* = 0.016).

### 2.5. B and T cell Subpopulations Distribution at 6 and 12 Months after Kidney Transplantation in Subclinical AbMR Patients

As it is well known, induction therapy causes significant cell depletion. However, this effect in contrast to CD4^+^ and CD8^+^ T cells does not affect CD19^+^ B cells.

Regarding what we have seen when pretransplant B cell subpopulations were studied, patients with subclinical AbMR presented a reduction in absolute numbers of transitional T2 cells at 6 months (0.21; IQR 0.08–1.24 cells/µL) and 12 months (0.38; IQR 0.11–0.89 cells/µL) when compared with the non-rejection group (1.11; IQR 0.72-2.24 cells/µL and 1.38; IQR 0.87–2.40 cells/µL), *p* = 0.067 and *p* = 0.005, respectively.

**Figure 8:**
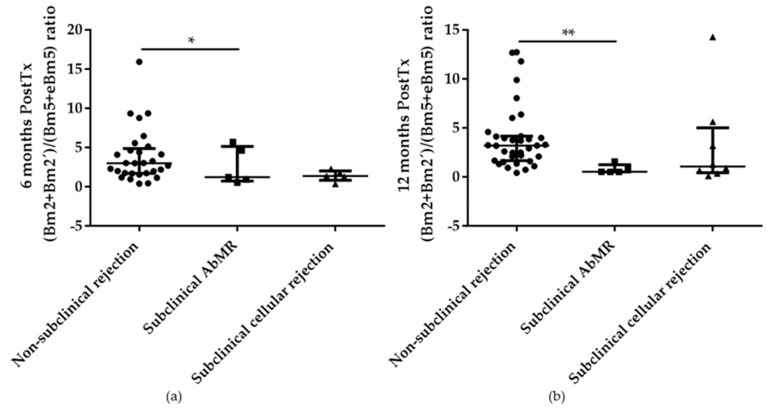
Significant reduction of active/memory B cells ratio between patients with subclinical antibody-mediated rejection (AbMR; *n* = 5) detected retrospectively in the surveillance biopsy performed at 1 year after kidney transplantation, and non-rejection patients (*n* = 38) at 6 months (**a**) and 12 months (**b**) after kidney transplantation. * indicates *p* < 0.05, and ** *p* < 0.01

A tendency to an increase in memory B cell subpopulations was also detected at 6 months and it was confirmed at 12 months after KT where a statistically significant reduction in the naïve/memory B cells ratio and (Bm2 + Bm2’)/(Bm5 + eBm5) ratio were observed in patients with subclinical AbMR in comparison with non-rejection KT patients, *p* = 0.05 and *p* = 0.002, respectively (Figure 8a,b).

At 12 months after kidney transplantation no differences were observed in T cells subpopulations between the analyzed groups. Nevertheless, at 6 months posttransplantation an increase in CD4^+^ TEMRA cells and therefore, a reduction in CD4^+^ naïve/TEMRA ratio was observed in patients with subclinical AbMR when compared with non-rejection group, *p* = 0.012. 

## 3. Discussion

Unlike the results showed by different groups where no differences were found in BAFF levels between kidney transplant recipients and controls [31] indicating that this molecule is not a prognostic marker for allograft dysfunction, or that no correlation exists between BAFF and the production of DSA before and after transplantation [32], in the present study we observed that patients with AbMR presented higher levels of BAFF before transplantation, being also elevated BAFF serum levels in those patients with anti-HLA antibodies and DSA. These results are in a relationship published by other authors showing that BAFF is associated with allograft survival [37], acute antibody-mediated rejection as well as the presence of DSA [28,29,38]. A meta-analysis published recently also corroborates our results, indicating that the incidence of antibody-mediated rejection is higher in those patients with higher levels of serum BAFF. BAFF levels are higher in patients with anti-HLA antibodies too [34]. Taking all of this into account and considering the results that we have obtained, we suggest that pretransplant serum BAFF levels could be an important non-invasive biomarker for the prediction of the development of antibody-mediated rejection, independently of classical variables. Even though the effect of an increase of 1 pg/mL of BAFF levels is limited, an increase of 100 pg/mL of BAFF levels increases 1.35 times the risk of developing antibody-mediated rejection during the first years after kidney transplantation, which is not negligible. In addition, after stratifying kidney transplant patients in high and low groups according to their pretransplant serum BAFF levels, the effect of having higher pretransplant BAFF levels increased around seven times the risk of developing antibody-mediated rejection during the first 12 months after kidney transplantation. We also observed that serum BAFF levels, measured at 6 and at 12 months after transplantation were also higher in those patients with subclinical antibody-mediated rejection detected in the surveillance biopsy, corroborating the possible contribution of BAFF to the pathogenesis of antibody-mediated graft damage. Considering this aspect, Won Min et al. described that while pretransplant BAFF levels showed significant association with early rejection, posttransplant BAFF levels measured at the time of indication biopsy are not associated with allograft rejection [25].It is important to highlight some relevant aspects of our study such as the prospective monitoring of the kidney transplant patients at 6 and at 12 months after kidney transplantation, which differentiates it from other transversal studies or with a shorter period of follow-up [28,29]. In the same way we would like to emphasize the importance of surveillance biopsy that gives up data about the possible existence of a subclinical rejection. To our knowledge this is the first study that combines the monitoring of serum BAFF levels and B and T cell subpopulations in order to study their association with the development of antibody-mediated rejection in kidney transplant patients.

Another relevant aspect is the well-established role of BAFF in the development of autoimmune diseases. It is known that elevated levels of serum BAFF in patients with autoimmune diseases correlate with the severity of the disease [39,40,41,42], as well as with levels of pathogenic autoantibodies [40,43,44]. Therefore, it could be possible to establish an interrelationship between these two different processes, the autoimmune response and the alloresponse in transplantation. BAFF could be an important player in the pathogenesis of both processes, promoting the production of autoantibodies and anti-HLA antibodies, respectively, and with that the outbreak of the autoimmune and rejection processes.

In order to avoid both mentioned situations, tolerance mechanisms are essential and B cells play an important role on them. Several B cell features have been associated with the occurrence of operational tolerance in kidney transplant recipients. Among these features, it should be highlighted that tolerant patients display a redistribution of B cell subsets, with a decrease in memory cells and an increase in transitional and naïve B cells, as well as a gene expression pattern dominated by B cell related genes, that can allow us to discriminate acute kidney allograft rejection from stable graft function [45,46,47]. In the present study, and in agreement with other publications [48], we observed that kidney transplant patients who suffered antibody-mediated rejection, opposite to tolerant patients, presented lower levels of pretransplant transitional and naïve B cells and higher levels of memory B cells. Therefore, antibody-mediated rejection patients showed reduced levels of transitional B cells, which are described as regulatory cells [49], and increased levels of switched memory B cells that present a strong antigen-presenting capacity and are mainly involved in antibody production. This aspect can be facilitated by the presence of high levels of serum BAFF in this type of patient, which contributes to the survival and differentiation of mature B cells. The promotion of a mature B cell reaction is also dependent on T–B cell cooperation and, accordingly, our data point for a balance toward a more mature phenotype in CD4^+^ T cells.

However, there are some limitations in our study. Despite being similar figures of patients with antibody-mediated rejection to those described in literature, the inclusion of more patients would be needed. In the same way, analysis of samples at multiple time points between transplantation and the appearance of the clinical event could be necessary in order to determine a better knowledge of the dynamics of B cell subpopulations. 

In conclusion, in this study we established together the role of BAFF and B cell subsets before and after transplantation in the development of antibody-mediated rejection, proposing that higher BAFF levels as well as an increase in the memory B cell repertoire contribute to the pathogenesis of antibody-mediated graft damage and could be considered as an independent non-invasive biomarker for predicting and diagnosis of rejection.

## 4. Materials and Methods 

### 4.1. Patients

The study was conducted following the rules of Declaration of Helsinki and approved by the Regional Ethics Committee in our Institution (reference number: 2014/161; 1 August 2014). A total of 109 consecutive kidney transplants performed in HUMV from February 2015 to February 2018 were recruited for the study after given written consent prior kidney transplantation. KT patients treated with rituximab before kidney transplantation were excluded from the study. The main demographic, immunological, and clinical parameters are summarized in Table 1 and Supplementary Table S1. The patients were prospectively monitored prior KT and subsequently 6 and 12 months after KT. Indication graft biopsies were performed due to the presence of a sustained delayed graft function, an increase of serum creatinine or proteinuria development. Surveillance biopsies were routinely carried out at one-year after transplantation. All the biopsies performed were reclassified according to the Banff 2017 criteria. Antibody mediated rejection was diagnosed following the Banff criteria [50]. Forty sex–age matched healthy subjects (HS) were tested for immunological parameters as control.

Surveillance biopsy was routinely performed at one year after KT in 70 patients after given written informed consent.

For the prospective analysis at 6 and 12 months after KT, 51 patients without clinical rejection during the first 12 months, which did not received any treatment (thymoglobulin, rituximab, plasmapheresis, or intravenous immunoglobulin) apart from induction therapies and maintenance immunosuppressive treatments, and with a surveillance biopsy that showed the possibility of the development of a subclinical AbMR were included. Results from surveillance biopsies showed that five of these patients (9.8%) developed a subclinical AbMR.

### 4.2. ELISA serum BAFF levels

The BAFF serum levels were measured by ELISA (R&D Systems, Minneapolis, MN, USA) following the manufacturer instructions. The sensitivity for BAFF serum levels was 62.5–4000 pg/mL.

### 4.3. Anti-HLA Antibodies and DSA

The presence of anti-HLA antibodies was tested using LABScreen Single Antigen Class I and II (One Lambda Inc., Canoga Park, CA, United States), according to manufacturer’s instructions, and analyzed on a Luminex platform (LabScan100, One Lambda Inc., Canoga Park, CA, USA).

HLA typing was performed for HLA locus A, B, C, DRB1, and BQB1 in all patients using sequence-specific oligonucleotide probes (SSOP) and in all donors by low resolution sequence-specific primers (SSP; One Lambda Inc., Canoga Park, CA, United States).

DSA was defined as the anti-HLA antibodies of the recipient corresponding with HLA types of the donor.

### 4.4. Flow Cytometry for B Cell Subsets

**Figure 9:**
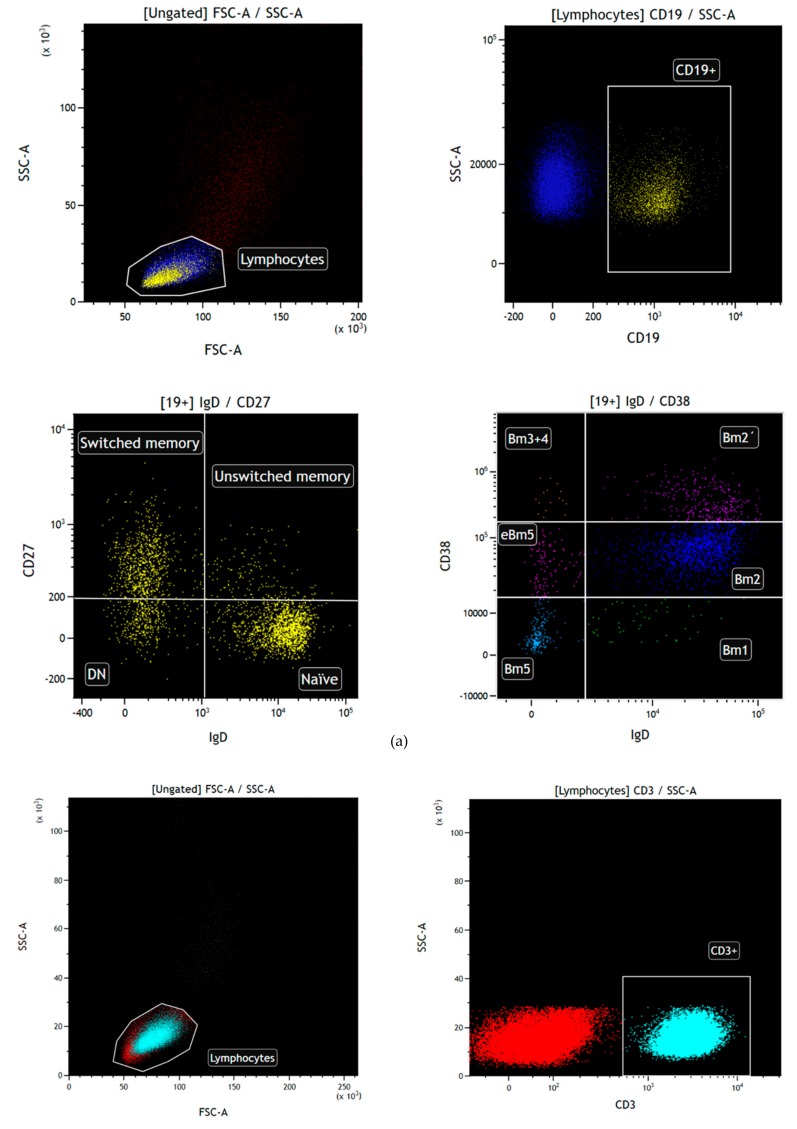

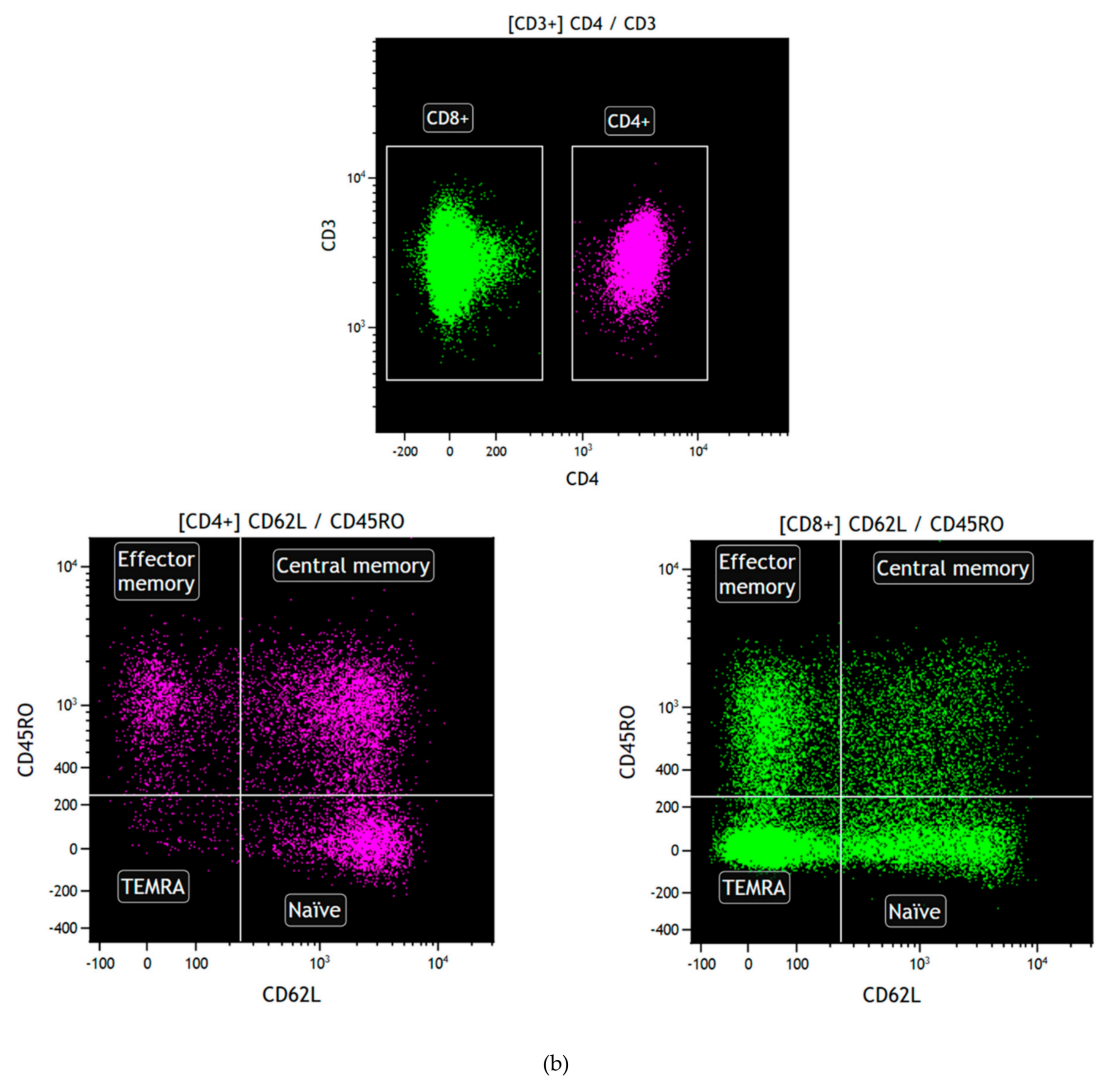
(**a**). Gating strategy of B cell subpopulations. Lymphocytes were identified by FSC (forward scatter) and SSC (side scatter). From lymphocyte population, CD19^+^ cells were selected, and based on them and according to IgD and CD27 markers, naïve (IgD^+^ CD27^-^), unswitched memory (IgD^+^ CD27^+^), and switched memory (IgD^-^ CD27^+^) B cells were identified. Furthermore, based on CD19^+^ cells and according to IgD and CD38 markers, Bm1 (IgD^+^ CD38^-^), Bm2 (IgD^+^ CD38^+^), Bm2’ (IgD^+^ CD38^high^), Bm3Bm4 (IgD^-^ CD38^high^), eBm5 (IgD^-^ CD38^+^), and Bm5 (IgD^-^ CD38^-^) cells were identified. Transitional type 2 cells were selected using a different gating strategy in order to obtain a pure population. For this gating strategy, CD19^+^ lymphocytes were selected, and from them those cells CD10^+^, CD20^+^, CD21^+^, CD5^-^, CD24^+^, and CD38^+^. (**b**). Gating strategy of CD4^+^ and CD8^+^ T cell subpopulations. Lymphocytes were identified by FSC (forward scatter) and SSC (side scatter). From lymphocyte population, CD3^+^ cells were selected, and based on them, CD4^+^ and CD8^+^ cells. Since CD4^+^ and CD8^+^ cells, and according to CD62L and CD45RO markers, naïve (CD62L^+^ CD45RO^-^), central memory (CD62L^+^ CD45RO^+^), effector memory (CD62L^-^ CD45RO^+^), and TEMRA (CD62L^-^ CD45RO^-^) T cells were identified.

Peripheral blood samples were freshly stained and processed following standard procedures, as previously described [51]. The following monoclonal antibodies were: anti-CD27-fluorescein isothiocyanate (FITC) clone M-T271, CD138-FITC clone MI15, CD24-phycoerythrin (PE) clone ML5, CD268-PE clone 11C1 and IgM-allophycocyanin (APC) clone G20-127 (BD Biosciences, San Diego, CA, USA), CD19-phycoerythrin-cyanine 5.5 (PC5.5) clone J3-119 (Beckman Coulter, Brea, CA, USA), CD38-PE cyanine 7 (Cy7) clone HIT2, CD5-APC clone UCHT2, CD10-APC Cy7 clone HI10a, CD25-Pacific Blue clone BC96 and IgD-Brilliant Violet 510 clone IA6-2 (BioLegend, San Diego, CA, USA) and CD27-APC Vio770 clone M-T271, CD21-VioBlue clone HB5, and CD20-VioGreen clone LT20 (Miltenyi Biotec, Bergisch Gladbach, Germany), to identify different B cell subsets. The following monoclonal antibodies were used to T cell subpopulations identification: anti-CD62L-FITC clone DREG56 (Beckman Coulter, Brea, CA, USA), CD45RO-PE clone UCHL1 (BD Biosciences, San Diego, CA), CD28-PC5.5 clone L293, CD27-PE Cy7 Vio770 clone 1A4CD27, CCR7-APC clone REA108 (Miltenyi Biotec, Bergisch Gladbach, Germany), CD4-APC Vio770 clone VIT4, and CD3-VioBlue clone UCHT1 (Immunostep, Salamanca, Spain). The immunophenotype for B and T cell subsets identification were performed as described [52] and are detailed in Supplementary Tables S2 and S3. Gating strategy used for the different B and T cell subpopulations selection is described in Figure 9. Percentages of the different B and T cell subpopulations before transplantation and at 6 and 12 months after kidney transplantation in each of the groups are described in Supplementary Tables S4 and S5.

### 4.5. Statistical Analysis

Statistical analysis was performed using SPSS v.22.0 (IBM Corp., Armonk, NY, USA) and Graph Pad Prism software. The distribution of continuous variables was assessed using Kolmogorov–Smirnov/Shapiro–Wilk tests where indicated. Results were expressed as mean ± standard deviation or median + interquartile range (IQR) for continuous variables and percentages for categorical data. Comparisons were based on the chi squared test for categorical data and Mann–Whitney test for nonparametric continuous data. Within-group comparison of quantitative variables was undertaken using the Wilcoxon matched-pair test. Spearman rank correlation was used to quantify associations between continuous variables. Receiver operating characteristic (ROC) analysis and Youden’s index were used to determine the optimal cut-point with higher sensitivity and specificity. Rejection free survival was tested by the Kaplan–Meier survival test. Cox and multivariate regression analysis was performed to assess the independent role of the studied variables in the development of AbMR. Collinearity between variables included in the multivariate analysis was also tested. A two-sided *p* value ≤0.05 was considered statistically significant. In the figures * indicates *p* < 0.05, ** *p* < 0.01, and *** *p* < 0.001.

## Figures and Tables

**Table 1:** Demographic, clinical, and immunological parameters of kidney transplant patients.

	ESRD (*n* = 109)			
	*n*	Mean / Median	SD / IQR	%
Recipient age (years)	109	55	43–62	
Recipient gender (male %)				57.8
Etiology of ESRD^1^:				
Glomerular	37			33.9
Diabetes mellitus	28			25.7
PKD	16			14.7
Interstitial	9			8.3
Vascular	8			7.3
Non-filiated	7			6.4
Other causes	4			3.7
Retransplantation	23			21.1
Hypersensitized (>90%)	7			6.4
PreTx Anti-HLA Ab	33			30.3
PreTx DSA	3			2.8
Donor age (years)		52	44–62	
DGF	26			23.9
CIT (hours)		17	9–21	
Induction therapy:	78			71.6
Thymoglobulin	53			67.9
Basiliximab	25			32.1
Biopsy C4d+	13			59.1
Biopsy g+ptc ≥ 2	18			81.8
Clinical rejection	22			20.2
Clinical AbMR	11			10.1
HLA-A Mismatches		1.21	0.63	
HLA-B Mismatches		1.50	0.62	
HLA-C Mismatches		1.37	0.62	
HLA-DRB1 Mismatches		1.32	0.67	
HLA-DQB1 Mismatches		1.04	0.67	

ESRD: end-stage renal disease; PKD: polycystic kidney disease; DGF: delayed graft function; CIT: cold ischemia time; AbMR: antibody-mediated rejection; PreTx: pre transplantation.

## Supplementary Materials

Supplementary materials can be found at https://www.mdpi.com/1422-0067/21/3/779/s1.

## Abbreviations

AbMR	Antibody-mediated rejection
DSA	Donor-specific antibodies
BAFF	B-cell activating factor
BAFFR	BAFF receptor
KT	Kidney transplantation
PRA	Panel-reactive antibodies
IQR	Interquartile range
SD	Standard deviation
HS	Healthy subjects
cPRA	Calculated-panel reactive antibodies
ROC	Receiver operational curve
AUC	Area under curve
CI	Confidence interval
Tx	Transplantation
CIT	Cold ischemia time
DGF	Delayed graft function
HR	Hazard ratio
ESRD	End stage renal disease
PKD	Polycystic kidney disease
PreTx	Pre-transplantation
PostTx	Post-transplantation
SSOP	Sequence-specific oligonucleotide probes
SSP	Sequence-specific primers
FITC	Fluorescein isothiocyanate
PE	Phycoerythrin
APC	Allophycocyanin
Cy5.5	Cyanine 5.5
Cy7	Cyanine 7
