# Supplementary material for: High Pretransplant BAFF Levels and B-cell Subset Polarized towards a Memory Phenotype as Predictive Biomarkers for Antibody-Mediated Rejection
Source: Int J Mol Sci. 2020 Jan 25;21(3):779. doi: 10.3390/ijms21030779 (PMC7037386; doi:10.3390/ijms21030779)
Supplement: Supplementary file 1 [file ijms-21-00779-s001.zip › Supplementary Table 1.pdf]

Supplementary Table 1. Demographic, clinical and immunological parameters of AbMR KT patients vs. non-rejection group

	**Non-rejection (n=87)**	**AbMR (n=11)**	***p***
Recipient age (years)	56 (43-63)	51 (44-63)	0.875
Recipient gender (male)	52 (59.8%)	5 (45.5%)	0.364
Etiology of ESRD:			
Glomerular	30 (34.5%)	3 (27.3%)	0.634
Diabetes mellitus	19 (21.8%)	3 (27.3%)	0.684
PKD	15 (17.2%)	1 (9.1%)	0.491
Interstitial	6 (6.9%)	2 (18.2%)	0.198
Vascular	6 (6.9%)	2 (18.2%)	0.198
Non-filiated	7 (8.0%)	0	
Other causes	4 (4.6%)	0	
Retransplantation	17 (19.5%)	3 (27.3%)	0.549
Hypersensitized (>90%)	4 (4.6%)	3 (27.3%)	0.006
PreTx anti-HLA Ab	27 (31%)	5 (45.5%)	0.337
Donor age (years)	53 (44-62)	55 (48-64)	0.408
DGF	16 (18.4%)	7 (63.6%)	0.001
CIT (hours)	16 (9-21)	21 (21-23)	0.012
Induction therapy:			
Thymoglobulin	41 (47.1%)	5 (45.5%)	0.917
Basiliximab	19 (21.8%)	5 (45.5%)	0.086
Biopsy C4d+	0	10 (90.9%)	
Biopsy g+ptc ≥ 2	0	10 (90.9%)	
HLA-A Mismatches	1.18 (0.64)	1.18 (0.60)	0.959
HLA-B Mismatches	1.52 (0.63)	1.36 (0.67)	0.418
HLA-C Mismatches	1.37 (0.61)	1.36 (0.67)	0.980
HLA-DRB1 Mismatches	1.30 (0.68)	1.36 (0.67)	0.786
HLA-DQB1 Mismatches	1.05 (0.65)	1.00 (0.89)	0.861

ESRD: end-stage renal disease; PKD: polycystic kidney disease; DGF: delayed graft function; CIT: cold ischemia time; AbMR: antibody-mediated rejection; PreTx: pre transplantation.
