# Supplementary material for: High Pretransplant BAFF Levels and B-cell Subset Polarized towards a Memory Phenotype as Predictive Biomarkers for Antibody-Mediated Rejection
Source: Int J Mol Sci. 2020 Jan 25;21(3):779. doi: 10.3390/ijms21030779 (PMC7037386; doi:10.3390/ijms21030779)
Supplement: Supplementary file 1 [file ijms-21-00779-s001.zip › Supplementary Table 2.pdf]

Supplementary Table 2. Immunophenotype for B cell subset identification

B cell subset	CD markers
Follicular B	CD19+CD10+CD20+CD21+CD5high
Marginal zone	CD19+CD20+CD21+CD24highCD38-
Pre B1	CD19+CD10+CD20-
Pre B2	CD19+CD10+CD20+CD21+CD24+CD38high
Transitional B T1	CD19+CD24+CD38+CD5high
Transitional B T2	CD19+CD24+CD38+CD5low
Bm1	CD19+IgD+CD38-
Bm2	CD19+IgD+CD38+
Bm2´	CD19+IgD+CD38high
Bm3Bm4	CD19+IgD-CD38high
eBm5	CD19+IgD-CD38+
Bm5	CD19+IgD-CD38-
Naïve	CD19+IgD+CD27-
Unswitched-memory B	CD19+IgD+CD27+
Switched-memory B	CD19+IgD-CD27+
Plasma cells	CD19-CD20-CD27highCD38highCD138+
