# Supplementary material for: High Pretransplant BAFF Levels and B-cell Subset Polarized towards a Memory Phenotype as Predictive Biomarkers for Antibody-Mediated Rejection
Source: Int J Mol Sci. 2020 Jan 25;21(3):779. doi: 10.3390/ijms21030779 (PMC7037386; doi:10.3390/ijms21030779)
Supplement: Supplementary file 1 [file ijms-21-00779-s001.zip › Supplementary Table 3.pdf]

Supplementary Table 3. Immunophenotype for T cell subset identification

T cell subset	CD markers
CD4 naïve	CD3+CD4+CD62L+CD45RO-
CD4 central memory	CD3+CD4+CD62L+CD45RO+
CD4 effector memory	CD3+CD4+CD62L-CD45RO+
CD4 TEMRA	CD3+CD4+CD62L-CD45RO-
CD8 naïve	CD3+CD8+CD62L+CD45RO-
CD8 central memory	CD3+CD8+CD62L+CD45RO+
CD8 effector memory	CD3+CD8+CD62L-CD45RO+
CD8 TEMRA	CD3+CD8+CD62L-CD45RO-
