# Supplementary material for: High Pretransplant BAFF Levels and B-cell Subset Polarized towards a Memory Phenotype as Predictive Biomarkers for Antibody-Mediated Rejection
Source: Int J Mol Sci. 2020 Jan 25;21(3):779. doi: 10.3390/ijms21030779 (PMC7037386; doi:10.3390/ijms21030779)
Supplement: Supplementary file 1 [file ijms-21-00779-s001.zip › Supplementary Table 4.pdf]

Supplementary Table 4. Percentages of different B and T cell subpopulations before kidney transplantation in healthy subjects (n = 40), kidney transplant patients without clinical rejection (n = 87) and kidney transplant patients that developed antibody-mediated rejection (AbMR) (n = 11) during the first 12 months after kidney transplantation.

	**Healthy subjects (n=40)**	**Non-clinical rejection (n=87)**	**Clinical AbMR (n=11)**
Transitional B T2	4.87 (3.11-6.74)	1.34 (0.40-2.57)	0.15 (0.09-0.92)
Naïve B	60.77 (49.19-67.62)	59.33 (43.99-76.90)	27.30 (13.50-33.31)
Unswitched-memory B	16.36 (11.77-23.38)	10.08 (6.32-17.36)	6.25 (4.71-11.34)
Switched-memory B	16.04 (12.85-21.07)	18.89 (9.66-27.32)	44.33 (9.26-59.36)
Bm2	45.13 (39.39-53.48)	51.99 (41.18-61.55)	21.63 (4.56-29.27)
Bm2´	6.18 (4.35-9.44)	5.92 (3.37-10.55)	6.30 (2.06-11.36)
Bm5	11.17 (8.15-14.26)	13.28 (8.28-18.69)	8.04 (6.03-20.10)
eBm5	9.38 (7.18-13.15)	8.83 (4.99-12.79)	9.85 (4.94-15.81)
Naïve CD4	58.33 (39.66-66.58)	42.47 (23.46-59.70)	29.99 (15.49-38.78)
TEMRA CD4	0.50 (0.26-1.11)	3.29 (1.40-7.78)	12.90 (4.71-17.95)
Naïve CD8	51.97 (36.14-61.56)	40.02 (26.48-57.31)	25.50 (9.30-38.18)
TEMRA CD8	15.22 (9.32-30.77)	31.58 (21.73-53.34)	47.24 (34.38-62.85)

AbMR: antibody-mediated rejection
