# Supplementary material for: High Pretransplant BAFF Levels and B-cell Subset Polarized towards a Memory Phenotype as Predictive Biomarkers for Antibody-Mediated Rejection
Source: Int J Mol Sci. 2020 Jan 25;21(3):779. doi: 10.3390/ijms21030779 (PMC7037386; doi:10.3390/ijms21030779)
Supplement: Supplementary file 1 [file ijms-21-00779-s001.zip › Supplementary Table 5.pdf]

Supplementary Table 5. Percentages of different B and T cell subpopulations at 6 and 12 months after kidney transplantation in kidney transplant patients without subclinical rejection (n = 38) and kidney transplant patients that presented subclinical cellular (n = 8) and subclinical antibody-mediated rejection (AbMR) (n = 5) in the surveillance biopsy performed at 1 year after kidney transplantation.

	**Non-subclinical rejection (n=38)**	**Subclinical cellular rejection (n=8)**	**Subclinical AbMR (n=5)**
**Transitional B T2**			
6 months	1.19 (0.64-2.34)	1.07 (0.16-1.42)	0.22 (0.09-0.58)
12 months	1.58 (0.80-2.75)	1.16 (0.48-1.40)	0.48 (0.29-0.50)
**Naïve B**			
6 months	59.67 (31.74-72.42)	46.15 (25.79-59.24)	66.02 (24.45-79.29)
12 months	64.43 (47.05-77.16)	70.71 (38.78-72.39)	30.27 (23.62-54.71)
**Unswitched-memory B**			
6 months	10.27 (6.51-15.29)	9.54 (7.43-12.92)	6.17 (1.51-13.92)
12 months	13.01 (8.95-17.41)	16.54 (7.31-19.82)	6.32 (4.69-11.20)
**Switched-memory B**			
6 months	12.34 (5.88-16.92)	24.23 (15.48-29.69)	7.87 (3.62-25.56)
12 months	13.43 (6.89-20.80)	22.87 (11.24-34.62)	42.61 (22.89-48.56)
**Bm2**			
6 months	45.75 (25.91-57.25)	41.77 (13.63-57.04)	48.78 (27.84-71.28)
12 months	47.95 (34.68-57.16)	36.78 (21.11-67.18)	30.14 (27.32-58.36)
**Bm2´**			
6 months	3.88 (1.92-10.47)	4.31 (2.96-4.78)	1.61 (0.46-5.02)
12 months	4.75 (2.65-11.42)	2.63 (1.49-7.39)	0.71 (0.46-9.36)
**Bm5**			
6 months	8.91 (4.19-19.30)	17.25 (8.18-28.47)	13.03 (8.54-60.03)
12 months	8.43 (6.29-12.10)	19.65 (7.03-43.96)	41.00 (31.27-48.65)
**eBm5**			
6 months	9.39 (5.23-11.58)	15.30 (9.62-19.09)	7.82 (3.30-8.29)
12 months	8.23 (5.69-10.53)	13.31 (7.61-16.09)	17.47 (7.95-23.82)
**Naïve CD4**			
6 months	48.39 (18.11-60.87)	19.37 (4.95-63.95)	33.17 (11.82-50.45)
12 months	34.28 (15.51-52.40)	28.63 (15.99-55.20)	40.08 (18.73-61.18)
**TEMRA CD4**			
6 months	2.04 (1.07-5.81)	4.25 (1.02-7.08)	12.91 (4.93-19.88)
12 months	2.10 (1.02-7.57)	3.31 (2.24-10.79)	3.50 (1.18-8.09)
**Naïve CD8**			
6 months	30.14 (21.24-48.69)	20.54 (13.25-56.89)	24.61 (11.58-29.92)
12 months	32.46 (19.84-44.24)	46.18 (13.87-60.38)	37.91 (22.35-42.98)
**TEMRA CD8**			
6 months	46.79 (28.49-59.73)	43.44 (19.65-74.60)	54.19 (51.36-66.72)
12 months	48.20 (27.29-65.30)	40.29 (28.71-72.59)	46.27 (41.52-58.90)

AbMR: antibody-mediated rejection
